# Mortality in a pediatric secondary-care hospital in post-conflict Liberia in 2009

**DOI:** 10.1590/S1679-45082013000400002

**Published:** 2013

**Authors:** Thomaz Bittencourt Couto, Sylvia Costa Lima Farhat, Tony Reid, Cláudio Schvartsman

**Affiliations:** 1 Hospital das Clínicas, Faculdade de Medicina, Universidade de São Paulo, São Paulo, SP, Brazil; 2 Médecins sans Frontières – Operational Center Brussels, Brussels, BX, Belgium; 3 Hospital Israelita Albert Einstein, São Paulo, SP, Brazil; Hospital das Clínicas, Faculdade de Medicina, Universidade de São Paulo, São Paulo, SP, Brazil; Universidade de São Paulo, Universidade de São Paulo, Faculdade de Medicina, Hospital das Clínicas, São Paulo, SP, Brazil

**Keywords:** Causes of death, Mortality rate, Mortality, Hospital, pediatric, Child, Liberia, Shock, septic, Quality of health care

## Abstract

**Objective:**

To describe and analyze the causes of death in a pediatric secondary-care hospital (run by *Médecins sans Frontières*), in Monrovia, Liberia, 6 years post-civil war, to determine the quality of care and mortality in a setting with limited resources.

**Methods:**

Data were retrospectively collected from March 2009 to October 2009. Patient charts and laboratory records were reviewed to verify cause of death. Additionally, charts of patients aged over 1 month with an infectious cause of death were analyzed for decompensated septic shock, or fluid-refractory septic shock.

**Results:**

Of 8,254 admitted pediatric patients, 531 died, with a mortality rate of 6.4%. Ninety percent of deaths occurred in children <5 years old. Most deaths occurred within 24 hours of admission. The main cause of death (76%) was infectious disease. Seventy-eight (23.6%) patients >1 month old with infectious disease met the criteria for septic shock, and 28 (8.6%) for decompensated or fluid-refractory septic shock.

**Conclusion:**

Since the end of Liberia's devastating civil war, Island Hospital has improved care and mortality outcomes, despite operating with limited resources. Based on the available data, mortality in Island Hospital appears to be lower than that of other Liberian and African institutions and similar to other hospitals run by *Médecins sans Frontières* across Africa. This can be explained by the financial and logistic support of *Médecins sans Frontières*. The highest mortality burden is related to infectious diseases and neonatal conditions. The mortality of sepsis varied among different infections. This suggests that further mortality reduction can be obtained by tackling sepsis management and improving neonatal care.

## RESUMO

**Objetivo:**

Descrever e analisar as causas de morte em um hospital secundário pediátrico (administrado pelos Médicos sem Fronteiras) na Monróvia, Libéria, 6 anos após o fim da guerra civil, para determinar a qualidade dos cuidados e a mortalidade em uma realidade com recursos limitados.

**Métodos:**

Os dados foram coletados retrospectivamente de março a outubro de 2009. Os prontuários e dados laboratoriais foram revisados para verificar a causa de morte. Além disso, prontuários de pacientes com mais de 1 mês de vida com causa de morte infecciosa foram analisados, para avaliar a presença de choque séptico descompensado ou choque séptico refratário a fluidos.

**Resultados:**

Do total de 8.254 pacientes admitidos, 531 morreram, com taxa de mortalidade de 6,4%. Noventa por cento dos óbitos ocorreram em crianças <5 anos. A maioria das mortes ocorreu nas primeiras 24 horas de internação. A causa principal (76%) foi doença infecciosa. Apresentaram choque séptico 78 (23,6%) pacientes com mais de 1 mês de idade e doença infecciosa, e 28 (8,6%) apresentaram choque séptico descompensado ou refratário a fluidos.

**Conclusão:**

Desde o fim da devastadora guerra civil na Libéria, o *Island Hospital* tem melhorado a qualidade de cuidado e diminuído a taxa de mortalidade, apesar de atuar com recursos limitados. Com base nos dados disponíveis, a mortalidade do *Island Hospital* aparenta ser menor do que em outras instituições liberianas e africanas, e semelhante a de outros hospitais administrados pelos Médicos sem Fronteiras na África. A explicação para esse fato é o apoio logístico e financeiro dos Médicos sem Fronteiras. A maior carga de mortalidade está relacionada a doenças infecciosas e condições neonatais. A mortalidade por sepse variou entre diferentes infecções. Isso sugere que a mortalidade ainda pode ser reduzida ao se melhorarem o tratamento e os cuidados neonatais.

## INTRODUCTION

Located in West Africa, Liberia is a small country with an area of 111,370km^2^ and a population of approximately 3.5 million. By the end of the 14-year-long civil war, in 2003, 80% of the population had been displaced and most of the health infrastructure was in ruins^(1)^. In recent years, Liberia has transitioned into a stable country with a democratic government, despite its limited resources.

In Liberia, the mortality rate of children <5 years old in 2003 and 2004 was 235/1,000 live births, while the mortality in Africa, as defined by the World Health Organization (WHO), was 171/1,000 live births in 2003 and 167/1,000 live births in 2004^(2)^. In 2011, this number improved considerably, to 78/1,000, with a regional average of 101/1,000. The main causes of death in this age group in Liberia, according to WHO data in 2010, were neonatal causes (32%), malaria (18%), pneumonia (14%), measles (10%), and diarrheal diseases (9%)^(3)^.

The Millennium Development Goals of WHO aim to lower the infant mortality of Liberia (goal 4) to approximately 78 deaths/1,000 live births by 2015^(4)^, which already was achieved in 2011^(3)^.

For 2008, a publication founded by the WHO, UNICEF, and the Bill and Melinda Gates Foundation, estimated a total of 20,328 deaths in children <5 years old in Liberia. Of those, 6,425 and 13,904 deaths were for neonates and children aged 1 month to 59 months, respectively^(5)^.

Only 41% of people have access to health care in Liberia. The ratio of physicians is 0.03/1,000 inhabitants. Monrovia, the capital of Liberia, has an estimated 1.5 million inhabitants, which is nearly half of the population of the country^(2)^.

Monrovia has only one tertiary hospital, John Fitzgerald Kennedy Medical Center. Five secondary hospitals admit pediatric patients: Island, Benson, Eternal Love Winning Africa (ELWA), St. Joseph Catholic, and Redemption Hospital.

There is very little data on the pediatric mortality in post-conflict Liberia. In 2005, an analysis of patient records conducted on a now-closed referral hospital administered by *Médecins Sans Frontières – Operational Center Paris* (MSF-OCP), demonstrated a pediatric mortality rate of 13.1% for children admitted^(6)^.

In particular, the patterns of mortality have not been studied in post-conflict scenarios, where the emergency care of displaced individuals and trauma patients is replaced by many other problems, such as chronic malnutrition, infectious diseases, and household accidents. Liberia offered a unique opportunity to study pediatric mortality in a stable setting and in a well-run hospital, in a post-conflict area that still suffers from limited resources. Given the high burden of infectious disease in this kind of context, particular attention was paid to assessing septic shock.

## OBJECTIVE

To describe and analyze causes of death in a pediatric referral hospital in Monrovia, Liberia, 6 years post-civil war to determine the quality of care and the mortality rate in a stable setting with limited resources.

## METHODS

### Design

This was a retrospective, descriptive study of routine chart data from Island Hospital records from March 2009 to October 2009.

### Setting

The study was conducted in Island Hospital, which received severely-ill patients aged zero to 15 years. Patients were either from the immediate catchment area or referred by local clinics and hospitals.

All patients were triaged, and non-urgent cases were referred to a local clinic. Therefore, only patients who possibly required admission were seen in the emergency room.

At the time of the study, Island Hospital had 150 beds, divided as follows: emergency room (ER; 10 beds), Intensive Care Unit (ICU; 15 beds), Neonatal Ward (14 beds), Under 6-Months-Old Ward (24 beds), General Pediatrics (40 beds), Burn Ward (13 beds), and Chest Ward (34 beds).

The hospital was staffed and administered by *Médecins Sans Frontières – Operational Center Brussels* (MSF-OCB). Most of the clinical work was conducted by physician assistants (PAs). During weekdays, there was one PA in each ward, one in the ICU, and two in the ER. Overnight, there was one PA in the ICU and two in the ER. Three physicians (including one pediatrician) supervised the clinical work every weekday, and one physician was always on-call on nights and weekends. Every ward always had one nurse and one nurse aide, except for the ER and ICU, which were staffed with two nurses.

Despite the quantity and the severity of the conditions of the inpatients, few advanced life support resources were available. The hospital had intravenous crystalloids, a blood bank, antibiotics (including ceftriaxone), oxygen concentrators, bag-valve-masks, and intraosseous needles. However, it lacked continuous infusion pumps that would have allowed the use of vasopressors, ventilators to permit intubation, and supplies to establish central lines.

Equipment and medications were regularly supplied by MSF.

### Sample and data collection

Data was manually collected from hospital records. Patient charts, death certificates, and laboratory records of all patients that died in Island Hospital, from March to October 2009, were reviewed to determine the cause of death. In cases of death due to infectious diseases, the reviewer tried to classify it as decompensated septic shock or fluid-refractory septic shock. Other data collected included age, gender, presence of acute malnutrition, date of onset of disease before arrival at the hospital, date of admission, date of death, period of day (day or night), place of death (*e.g.*, ER or ICU), and time from admission to death.

In Island Hospital, diagnosis was mostly clinical, supported by a limited number of additional tests: Parachek (reliable, rapid *Plasmodium falciparum* test)^(7)^, blood smear, complete blood count, urinalysis, cerebrospinal fluid analysis and Pastorex^®^ for meningitis (latex agglutination test)^(8)^, Gram stain, tuberculin test (PPD), glucose levels, and digital X-ray. There was no capacity for blood, urine, or cerebrospinal fluid cultures.

Case definitions were based on the second edition of the WHO Pocket Book of Hospital Care for Children^(9)^, as follows:

– hypoxic ischemic encephalopathy due to perinatal asphyxia: neonate with history of lack of oxygen supply before, during, or immediately after birth, and convulsion, apnea, inability to suck, or poor motor tone;
– neonatal sepsis: neonate presenting with any risk signs (not feeding well, convulsions, drowsy or unconscious, movement only when stimulated or not at all, fast breathing >60/min, grunting, severe chest wall indrawing, central cyanosis, fever >38°C/100.4°F or hypothermia <35.5°C/95.9°F), or any other signs of severe bacterial infection (*e.g.*, deep jaundice, severe abdominal distension, signs of pneumonia, painful joints, joint swelling, reduced movement, many or severe skin pustules, umbilical redness, umbilical draining of pus, or bulging fontanelle);
– premature or low-birth-weight newborns:
  – neonates with a birth weight of 2 to 2.5kg or 35 to 36 weeks of gestation, if this information is available (usually strong enough to breastfeed and maintain their body temperature) AND
  – neonates with a birth weight of less than 2kg or less than 35 weeks, if this information is available (should be admitted to a special care unit);
– neonatal tetanus: neonate with irritability, difficulty in breastfeeding, trismus, muscle spasms and convulsions with onset at age of 3 to 14 days;
– pneumonia: cough or difficult breathing plus at least one of the following: fast breathing (≥50/min age 2 to 11 months, ≥ 40/min age 1 to 5 years), or lower chest wall indrawing. In addition, either crackles or pleural rub may be present on chest auscultation;
  – severe pneumonia: pneumonia with oxygen saturation <90% on pulse oximetry or central cyanosis, severe respiratory distress (*e.g.* grunting, very severe chest indrawing), inability to breastfeed or drink or vomiting everything, convulsions, lethargy or reduced level of consciousness, auscultatory findings of decreased or bronchial breath sounds or signs of pleural effusion or empyema;
– acute diarrhea: more than three loose stools/day;
– bloody diarrhea (dysentery): blood in stool associated with acute diarrhea;
– malaria: fever plus positive investigation (Paracheck or blood smear);
– meningitis: a history of fever and seizures with the presence of meningeal signs and altered consciousness plus positive cerebrospinal fluid analysis;
– severe malnutrion: weight for height <3 Z-scores of the median, presence of bilateral pitting edema, or middle upper arm circumference under 110mm;
– moderate malnutrion: weight for height between 2 and 3 Z-scores of the median;
– HIV: two positive HIV tests on children older than 18 months or one positive rapid test plus positive virological test;
– urinary tract infection: fever or urinary symptoms plus suggestive urine culture (if available) or analysis;
– tuberculosis: suspected in children with a history of unexplained weight loss, history of fever >2 weeks, chronic cough, and exposure to an adult with tuberculosis. Examination showing pleural effusion, non-tender adenopathy, signs of meningitis, especially if developed over several days with CSF containing mostly lymphocytes and elevated protein, abdominal swelling or progressive swelling or deformity of bone or joint, including spine. Positive Ziehl-Neelsen stain on sputum, suggestive chest X-ray, and PPD over 10mm;
  – according to local protocols, definitive diagnosis was considered with a Keith Edwards score ≥7^(10,11)^;
– the diagnosis of sepsis and septic shock in children over 1 month of age was as per the definition by American College of Chest Physicians/Society of Critical Care Medicine^(12–15)^;
– sepsis: suspected or proven infection and at least two of the following: fever (>38.5°C/101.3°F) or hypothermia (<36°C/96.8°F), tachycardia, tachypnea, and elevated or depressed leukocyte count;
– septic shock: sepsis and signs of decreased tissue perfusion, altered mental status, capillary refill time >2 seconds (cold shock) or flash capillary refill (warm shock), diminished (cold shock) or bouncing peripheral pulses, mottled cool extremities (cold shock), and urinary output <1mL/kg/h;
  – hypotension is not necessary for diagnosis, but when present defines decompensated shock with worse prognosis;
– fluid-refractory septic shock: septic shock unresponsive to adequate fluid resuscitation.

### Analysis

The collected data was entered into an Excel spreadsheet. Statistical analyses for frequencies and associations were performed using usando Statistical Package for the Social Sciences (SPSS), version 17.

### Ethics

This study met the MSF's Ethics Review Board-approved criteria for analysis of routinely collected program data. It was also submitted to ethical analysis by the *Hospital Israelita Albert Einstein* Ethics Committee, and approved in *Plataforma Brasil*, with the ethical presentation certificate (CAAE) number 14498713.7.0000.0071.

## RESULTS

From March 2009 to October 2009, 8,254 pediatric patients were admitted to Island Hospital and 531 died, with a mortality rate of 6.4%; of these, 62.9% died within the first 48 hours of admission. Fifty-five were brought dead to the hospital. These patients were not included on the total mortality rate since they were not actually admitted to the hospital.

The majority of deaths (90.4%) occurred in children <5 years old, and most patients who died were male (Table 1). A slight majority of patients died during nighttime hours. Deaths occurred almost exclusively in the ER and ICU departments.

**Table 1:** Distribution of mortality by gender, age and onset of illness

Characteristics		Death n (%)
Age		
	<1 month	147 (27.7)
	1 month-5 years	333 (62.7)
	>5 years	51 (9.6)
Gender		
	Male	300 (56.5)
	Female	231 (43)
Onset of disease*		
	<72 hours	209 (45.4)
	>72 hours	167 (36.3)
	Without information	84 (18.3)
Time from admission to death		
	<24 hours	174 (32.8)
	24-48 hours	160 (30.1)
	>48 hours-7days	110 (20.7)
	>7 days	87 (16.4)
	Average	4 days
Period of day**		
	Daytime	187 (48.7)
	Overnight	195 (50.8)
	Unknown	2 (0.5)
Location of fatality**		
	Emergency Room	185 (48.2)
	Intensive Care Unit	197 (51.3)
	Ward	2 (0.5)

* Registered from April to October; total of registers=460;

** registered from June to October; total of registers=384.

**Figure 1:**
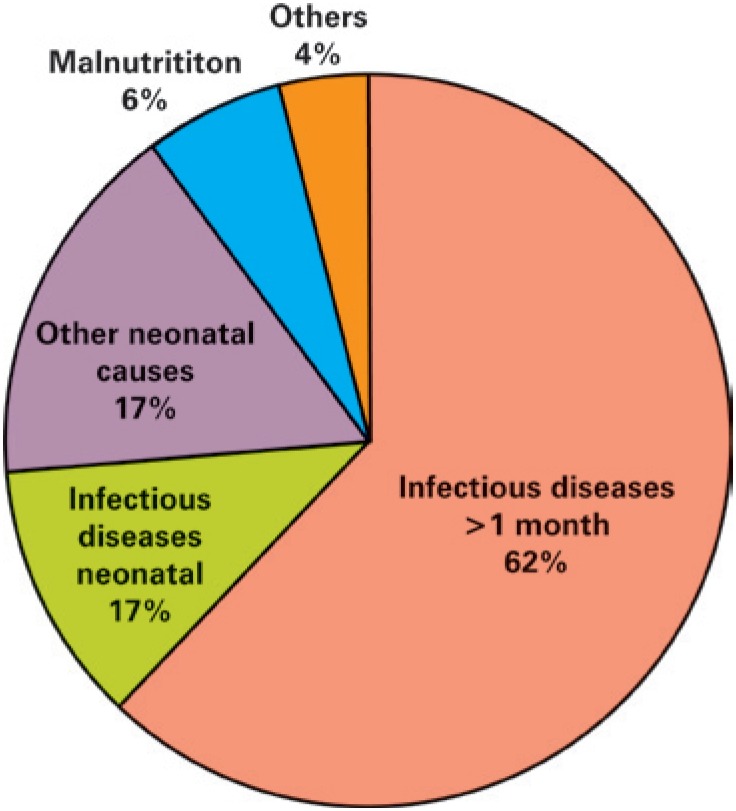
Main causes of death

The main cause of death was infectious diseases (73%; Figure 1). Patients older than 1 month died mainly of infectious diseases (86.2%) and burns (20 patients in total), most of whom (80%) died of secondary infection and 4 of non-infectious complications (Table 2).

**Table 2:** Main causes of death in children older than one month (n=384)

Cause of death		n (%)
Infectious diseases		331 (86.2)
	Malaria	105 (27.3)
	Pneumonia	95 (24.7)
	Diarrhea	40 (10.4)
	Tuberculosis	24 (6.3)
	Burns + secondary infection	16 (4.2)
	Aids	15 (3.9)
	Meningitis	11 (2.9)
	Other infections*	25 (6.5)
Malnutrition		31 (8.1)
Burns		4 (1)
Other		18 (4.7)
Dead on arrival		47**
Total		384 (100)

* Skin infections (6), hepatitis (6), urinary tract infection (2), tetanus (1), ear infection (1), fever of unknown origin (1), enteric fever (1), sepsis (4), or septic shock (3) with no clear source of infection;

** not counted in total.

In cases of infectious causes of death in children older than 1 month, 23.6% of patients met the criteria for septic shock and 8.6% for decompensated or fluid-refractory septic shock (Table 3).

**Table 3:** Presence of septic shock in children older than 1 month

Infectious diseases	n=331	Deaths (%)	Infectious diseases (%)
Septic shock	78	20.3	23.6
Decompensated or fluid-refractory septic shock	28	7.3	8.6

Malnutrition in children older than 1 month was observed in 113 (29.4% of patients older than 1 month); 83 (73.5%) children were severely malnourished and 30 (26.5%) were moderately malnourished. In 31 (27.4%) of all malnourished patients, this was considered the main cause of death, while in 82 (72.6%) it was a secondary diagnosis.

In children younger than 1 month, the majority of deaths were due to infectious diseases (40.1%). A significant amount of deaths (55.4%) were due to neonatal causes probably related to lack of assistance during the gestation and delivery: prematurity (31.6%), hypoxic ischemic encephalopathy (21.9%), and congenital malformation (1.9%) (Table 4).

**Table 4:** Main causes of death in children less than 1 month of age (n=147)

Main cause of death		n (%)
Infectious diseases		59 (40.1)
	Neonatal sepsis	49 (33.3)
	Neonatal meningitis	4 (2.7)
	Neonatal tetanus	3 (2)
	Diarrhea	2 (1.4)
	Urinary tract infection	1 (0.7)
Other neonatal causes		88 (59.9)
	Prematurity	49 (33.3)
	Hypoxic ischemic encephalopathy	34 (29.3)
	Congenital malformation	3 (2)
	Intoxication	1 (0.7)
	Burns	1 (0.7)
	Dead on arrival	8*
Total		147 (100)

* Not counted in total.

## DISCUSSION

The mortality of children at Island Hospital from March 2009 to October 2009 was mainly due to infectious diseases, followed by other neonatal causes, malnutrition, and burns, and all known problems were related to the lack of basic necessities, *e.g.*, food, sanitation, and primary healthcare.

Island Hospital's child mortality rate, during the study period, compared favorably with reports published in the last decade in many African hospitals. For instance, the pediatric mortality rates were substantially higher in Nigeria (10%)^(16)^, Zimbabwe (15%)^(17)^, and Mali (21%)^(18)^.

It is a similar rate compared to mortality reported in district hospitals in Kenya (4 to 15%, with mean of 7%)^(19)^ and other institutions managed by MSF (mean of 7%) in Niger (Dakoro, 7%; Guidam, 6%), Democratic Republic of Congo (Lubutu, 3%; Masisi, 9%), Sierra Leone (9%), Ethiopia (3%), Somalia (4%), and North Sudan (5%)^(20)^.

The 2009 mortality rate in Liberia was also lower than the 2005 rate. Huerga et al.^(6)^ described a 13% fatality rate among 1,509 children at Mamba Point Hospital in Liberia, which was administrated by MSF-Operational Center Paris. This mortality rate was similar to those observed at other hospitals in Monrovia during the same period: Redemption (11%), ELWA (14%), and Benson (17%) hospitals.

It is difficult to definitively know the reasons for the comparatively lower mortality rate at Island Hospital. In a recent publication describing mortality in eight hospitals run by MSF, the mortality rate, very similar to the average of this study, was attributed to standardization of treatment by MSF and resources mobilized by the organization^(20)^.

In fact, Island Hospital had some unique characteristics that could have a positive impact on mortality. First, it was an all children's hospital, with an organized triage^(21)^. Staff underwent regular training sessions and, as this was a pediatric hospital, they were experienced in treating critically ill children and had standard treatment protocols. Additionally, the staff was paid above average wages for Liberia. Finally, there were no supply problems for drugs and basic equipment. A study done in Guinea-Bissau with the introduction of standard treatment and financial support showed a reduction in malaria mortality by half (10 to 5%)^(22)^. This study supports the inference that conditions supplied by MSF allowed to have a relatively lower mortality.

A number of factors contributed to deaths at the Island Hospital. Many families had a difficult time traveling to the hospital. Long distances, lack of transportation, poor quality roads, and security issues all made it difficult to reach the healthcare unit in a timely manner. In other African institutions, the cost of care is unaffordable for many families^(23)^. Although care at Island Hospital was totally free-of-charge, not all families were aware of that fact. For some families, even small indirect costs were too much to bear, *e.g.*, some families could not afford to miss work to take to a sick child to the hospital. Additionally, some families distrusted Western medicine. The importance of delays in taking children in for treatment was seen by the average onset of disease, before seeking care, and the number of children who were dead on arrival.

The main causes of death in Island Hospital mirror those reported by WHO in 2006^(2)^, which reported that 29% of child deaths were from neonatal causes similar to our study's finding. Liberia has one of the worst levels of perinatal care in the world; the maternal mortality rate is 994/100,000^(1)^. Island Hospital received many neonates, most of whom had been delivered at home with little or no antenatal care. This made it very difficult to accurately determine the diagnosis of death. For instance, if a baby who had been delivered at home and had no clear antenatal history arrived at the hospital in cyanosis, it was nearly impossible to know whether the main problem was sepsis or hypoxic ischemic encephalopathy due to perinatal asphyxia.

Multiple diagnoses were listed for each child in the clinical history. The first most severe diagnosis was considered to be the cause of death. Although it was relatively easy to diagnose most diseases, for hypoxic ischemic encephalopathy due to perinatal asphyxia and sepsis, the criteria were harder to define, since both conditions share many clinical features. For instance, of all patients diagnosed with neonatal sepsis, only 2.6% were also diagnosed with hypoxic ischemic encephalopathy, but of the patients diagnosed with hypoxic ischemic encephalopathy, one third were classified as neonatal sepsis. This diagnostic difficulty is largely due to the lack of reliable perinatal data. Unfortunately, inadequate registration is known to occur in 98% of the world's 4 million neonatal deaths, highlighting the lack of reliable cause of death data in the setting where most deaths occur^(24)^.

Malaria was the leading cause of death in children older than 1 month. The hospital had a blood bank, and transfusions were given when anemia was a complication. In all cases of cerebral malaria, a lumbar puncture was done due to the difficulty of distinguishing malaria from bacterial meningitis^(25)^. Of the patients older than 1 month that had meningitis, over 25% also had malaria, reinforcing the need for cerebral spinal fluid analysis to differentiate the two conditions, which is consistent with recent WHO recommendations^(9)^.

The mortality of pneumonia in children older than 1 month in this study might be explained by several factors. First, case definition was based on the presence of tachypnea, which includes many other diagnoses in addition to pneumonia, such as bronchiolitis, wheezing, influenza, pertussis, asthma, and high respiratory rate due to acidosis, a fact already mentioned in other publications analyzing pneumonia mortality within this context^(26)^. Also, except for oxygen, respiratory support was lacking. Finally, the previously mentioned delayed arrival at the hospital in many patients also accounted for many severe respiratory presentations.

Diarrhea was also a major concern. This can be explained by the lack of basic sanitation; cholera was endemic, and malnourished children, of which there were a high number, are especially vulnerable to dehydration^(27)^.

Interestingly, although the recorded number of cases of septic shock was low, after analyzing charts of children older than 1 month, whose cause of death was infectious, and applying international criteria, this number rose significantly. This was expected, since most of the mortality was related to infectious diseases. This finding implies that the burden of sepsis is high and underestimated^(28)^.

Although many patients died from sepsis, they were often not classified as “septic shock” simply because the international definition was not applied. It was impossible to know if it was a decompensated shock, since many times the blood pressure was not measured. Fluid-refractory shock demands actually giving fluid bolus, a treatment that was not used in many cases. It is unknown how many of these patients would have responded to aggressive fluid therapy, or even if giving fluids would decrease mortality. A recent study conducted in limited resource settings, the FEAST trial, demonstrated higher 48-hour mortality with fluid bolus for septic shock in this context^(26)^.

In developed countries, neonatal and pediatric sepsis outcomes have improved with the advent of neonatal and pediatric intensive care (a reduction in mortality from 97% to 9%)^(13)^. With the additional implementation of “best clinical practices” of the American College of Critical Care Medicine Clinical Practice Parameters for Hemodynamic Support of Pediatric and Neonatal Shock, the mortality rates are estimated at zero to 5% in previously healthy children and at 10% in chronically ill children with septic shock^(12)^. This suggests that there is room to improve outcomes for treating sepsis at Island Hospital. However, implementing the quality of care available in developed countries, including Liberia, may not be feasible at this time given current economic conditions.

There were some limitations regarding this study. Finding hospitals equal to Island with which to compare mortality data was very difficult, and historic mortality data in Island Hospital was nonexistent.

Although all clinicians made a concerted effort to ensure good record keeping, in many charts the diagnostic information had to be reconstructed from notes made by several clinicians.

Basic communication was also a challenge. Although English is the official language of Liberia, many tribal languages exist. This led to two levels of possible miscommunication: verbal miscommunications between the patient and hospital staff and written communication by the staff when recording history in charts. Frequently, the study authors had a difficult time obtaining an accurate clinical history from the written notes, especially for the children who were dead on arrival and had no history recorded in their charts. Since autopsy was not possible, there was no way of confirming their actual cause of death.

## CONCLUSION

Since the end of Liberia's devastating civil war, Island Hospital has improved care and mortality outcomes, despite operating in a setting of limited resources. Based on the available data, the mortality rate at Island Hospital appears to be lower than that of other Liberian and African facilities, and similar to other *Médecins sans Frontières*-run hospitals across Africa. This can be explained by the financial and logistical support of *Médecins sans Frontières* that managed the hospital.

The highest mortality burden is related to infectious diseases and neonatal conditions. The mortality of sepsis was hidden among many different infections. This suggests that further mortality reduction can be obtained by tackling sepsis management and improving neonatal care.
